# Impact of adenomyosis on the prognosis of patients with endometrial cancer

**DOI:** 10.1002/ijgo.13818

**Published:** 2021-07-18

**Authors:** Diego Raimondo, Antonio Raffone, Antonio Travaglino, Manuela Maletta, Paolo Casadio, Marco Ambrosio, Anna Chiara Aru, Angela Santoro, Gian Franco Zannoni, Luigi Insabato, Antonio Mollo, Fulvio Zullo, Renato Seracchioli

**Affiliations:** ^1^ Division of Gynecology and Human Reproduction Physiopathology Department of Medical and Surgical Sciences (DIMEC IRCCS Azienda Ospedaliero‐Univeristaria di Bologna, S. Orsola Hospital University of Bologna Bologna Italy; ^2^ Gynecology and Obstetrics Unit Department of Neuroscience, Reproductive Sciences and Dentistry School of Medicine University of Naples Federico II Naples Italy; ^3^ Pathology Unit Department of Advanced Biomedical Sciences School of Medicine University of Naples Federico II Naples Italy; ^4^ Pathology Unit Department of Woman and Child Health Agostino Gemelli University Polyclinic Catholic University of the Sacred Heart Rome Italy; ^5^ Department of Medicine, Surgery and Dentistry "Scuola Medica Salernitana University of Salerno Baronissi, Salerno Italy

**Keywords:** endometrium, gynecology, malignancy, myometrium, oncology, tumor

## Abstract

**Background:**

Despite the high prevalence of adenomyosis in hysterectomy specimens of endometrial carcinoma (EC) patients, the relationship between adenomyosis and EC prognosis appears unclear.

**Objective:**

To assess the prognostic value of coexistent adenomyosis in patients with EC.

**Methods:**

A systematic review and meta‐analysis was performed by searching six electronic databases for studies reporting data on prognosis of EC patients with and without coexistent adenomyosis. Studies with patient selection based on prognostic factors were excluded. Pooled univariate hazard ratio (HR) analyses for overall survival (OS) and disease‐free survival (DRF) were performed, using EC patients without adenomyosis as a control group. For DFS, pooled multivariate HR analysis was also evaluable.

**Results:**

Three studies of 2505 EC patients (553 with and 1952 without adenomyosis) were included. Compared with EC patients without adenomyosis, EC patients with coexistent adenomyosis showed a pooled HR of 0.533 (CI 95%, 0.329–0.864) for OS at univariate analysis; 0.536 (CI 95%, 0.334–0.859) for DFS at univariate analysis; and 0.875 (CI 95%, 0.331–2.315) for DFS at multivariate analysis.

**Conclusion:**

In EC patients with coexistent adenomyosis, the risk of death is halved compared with EC patients without adenomyosis. However, the independence of this association needs to be verified in future studies.

## Abstract 2

The risk of death is halved in EC patients with coexistent adenomyosis compared with EC patients without adenomyosis, whereas the risk of EC recurrence does not appear to be affected.

## 1 INTRODUCTION

Endometrial carcinoma (EC) is the most frequent gynecological tumor in high‐resource countries and the fourth in women worldwide.^1^, ^2^, ^3^, ^4^, ^5^, ^6^, ^7^ Both incidence and number of deaths per year have increased in recent years.^1^, ^2^ Increase in incidence appears related to the increase in EC risk factors, such as obesity, metabolic syndrome, and causes of hyperestrogenic state,^8^ while the increase in number of deaths seems to be due to poorly reproducible pathologic risk stratification of patients.^9^, ^10^ Hence, a more accurate and tailored risk assessment able to direct patient management is needed.

In the future, integration among all available prognostic approaches, such as The Cancer Genome Atlas program (TCGA) and Proactive Molecular Risk Classifier for Endometrial Cancer (ProMisE) molecular signatures,^2^, ^7^, ^11^, ^12^, ^13^, ^14^, ^15^, ^16^, ^17^ classic histopathological factors (histotype, tumor grade, myometrial infiltration, lymph node involvement, lymphovascular space invasion), and clinical characteristics (age, body mass index, clinical stage, other diseases)^18^ might lead to individualized management of patients.

In this regard, adenomyosis appears to be one of the most frequent diseases associated with EC on hysterectomy specimens.^19^, ^20^ Adenomyosis is defined by the migration of glands and stroma from the basal layer of the endometrium to the myometrium.^21^ In a recent meta‐analysis,^22^ EC patients with coexistent adenomyosis showed a significantly increased overall survival (OS). On the contrary, no difference was found in terms of disease‐free survival (DFS). However, some studies included in the meta‐analysis only included EC patients with an endometrioid histotype.^22^ As the endometrioid histotype shows the best prognosis in EC patients,^17^, ^23^ the results from this meta‐analysis may be affected by this patient selection. The aim of the present systematic review and meta‐analysis was to assess the prognostic value of coexistent adenomyosis in patients with EC of any histotype.

## 2 MATERIALS AND METHODS

The study followed a protocol that a priori defined each review step and was reported according to the Preferred Reporting Items for Systematic Reviews and Meta‐analyses (PRISMA) statement.^24^ Each step was completed by two authors and disagreements were solved by discussion with the authors who supervised the study (PC, AM, LI, FZ, RS).

Several searches were performed in six electronic databases: MEDLINE, Google Scholar, Web of Science, Scopus, ClinicalTrials.gov, and the Cochrane Library. Databases were searched from their inception to December 2020 with several combinations of the following text words: “endometr*”; “cancer”; “carcinoma”; “neoplas*”; “malignancy”; “tumour”; “tumor”; “adenomyosis”; “myometr*”. We also screened the reference list from each eligible article.

All peer‐reviewed articles reporting data about prognosis of EC patients with and without coexistent adenomyosis were included. A priori defined exclusion criteria were literature reviews, case reports, and studies with patient selection based on prognostic factors.

The Methodological Index for Non‐Randomized Studies (MINORS) was followed for assessing the risk of bias within studies.^25^ Each included study was assessed in six applicable domains related to risk of bias: (1) aim (if the study aim was clearly stated); (2) patient selection (if all consecutive patients were included); (3) data collection (if a study protocol was a priori defined and followed for data collection); (4) study endpoints (if clear endpoints were adopted); (5) endpoint assessment (if endpoints were assessed through univariate and multivariate analyses); and (6) lost to follow‐up (if patients with no prognosis data were less than 5% of the total study population).

We judged the included studies at “low risk,” “unclear risk,” or “high risk” of bias in each domain based on whether data were “reported and adequate,” “not reported,” or “reported but inadequate,” respectively.

The PICO (Population, Intervention, Comparator, Outcomes) framework was followed for data extraction.^24^ “Population” in this study consists of EC patients. “Intervention” (or risk factor) consists of diagnosis of adenomyosis. “Comparator” consists of absence of adenomyosis. “Outcomes” were OS (primary outcome) and DFS (secondary outcome). OS was defined as the time interval between the date of surgery and death or the last date of follow‐up, while DFS was defined as the time interval between the date of surgery and the date of EC recurrence or the last date of follow‐up.

In the included studies, the association of adenomyosis with EC survival outcomes was assessed at univariate analysis by using the log‐rank test and the Kaplan–Meier method. Subsequently, the independent prognostic value of adenomyosis was assessed at multivariate analysis using the Cox proportional hazards model.

Hazard ratios (HR) were reported for each individual study and as a pooled estimate on forest plots, with 95% confidence interval (CI), for OS and DFS univariate analysis, and DFS multivariate analyses. EC patients without coexistent adenomyosis were considered as the reference. The random‐effects model of DerSimonian and Laird was adopted for all analyses.

The inconsistency index *I* ^2^ was adopted to quantify statistical heterogeneity among studies, as previously described.^26^, ^27^ Heterogeneity was judged as null for *I* ^2^ = 0%, minimal for 0% < *I* ^2^ < 25%, low for 25% < *I* ^2^ < 50%, moderate for 50% < *I* ^2^ < 75% and high for *I* ^2^ ≥ 75%.

Comprehensive Meta‐Analysis software (Biostat Inc) and Review Manager 5.3 (Copenhagen: The Nordic Cochrane Centre, Cochrane Collaboration, 2014) were used as software for data analysis.

## 3 RESULTS

A total of 4002 papers were found through electronic database searches. Of these, 1034 papers remained after duplicate removal, 753 after title screening, and 54 after abstract screening. After full‐text assessment, three papers were included in the qualitative and quantitative analyses^28^, ^29^, ^30^ (Figure S1).

All three included studies were designed as observational retrospective cohort studies (Table 1). The study population consisted of 2505 women with EC: 553 (22.1%) with coexistent adenomyosis and 1952 (77.9%) without adenomyosis. Mean age ranged from 52.7 to 59 years, and mean body mass index (BMI, calculated as weight in kilograms divided by height in meters squared) ranged from 25.2 to 35.8 kg. Of the included studies with extractable data, 63.8% of patients were menopausal and 12.8% were nulliparous. EC patients had an endometrioid histotype in 88.6% of cases, FIGO Stage I in 78.7%, deep myometrial infiltration in 33.6%, and lymphovascular space invasion in 30.2% (Table 2).

**TABLE 1:** Details of the included studies

Study	Country	Setting	Type of cohort	Period of endometrial cancer diagnosis	Patient selection
2014 Matsuo^28^	USA	Los Angeles County Medical Center, USA	Retrospective cohort	2000–2012	Consecutive
2017 Zhang^29^	China	Hebei general Hospital, China	Retrospective cohort	2008–2014	Consecutive
2018 Boonlak^30^	Thailandia	Srinagarind Hospital, Thailand	Retrospective cohort	2010–2016	Consecutive

**TABLE 2:** Details of populations included in the study

Study	Total patients (No).	Adenomyosis No. (%)		Age, mean ± SD (range)	BMI, mean ± SD	Postmenopausal No. (%)	Multiparity No. (%)	Endometrioid No. (%)	Nonendometrioid No. (%)	Stage I No. (%)	Stage II N (%)	Stage III N (%)	Stage IV N (%)	5‐year survival No. (%)
2014 Matsuo^28^	571	Yes	271 (47.4)	52.7 ± 9.6	35.8 ± 9.1	—	197 (76.7)	229 (84.5)	42 (15.5)	202 (74.8)	18 (6.7)	38 (14.1)	12 (4.4)	242 (89.2)
		No	300 (52.5)	52.7 ± 10.7	35.5 ± 10.7	—	187 (65.6)	247 (82.3)	53 (17.7)	193 (64.3)	32 (10.7)	48 (16.0)	27 (9.0)	234 (78.2)
2017 Zhang^29^	1584	Yes	150 (9.4)	53	27	81 (54)	—	140 (93.3)	10 (6.67)	139 (92.6)	11 (7.3)	137 (91.3)	13 (8.7)	138 (92.1)
		No	1434 (90.5)	55	26.6	930 (64.8)	—	1294 (90.2)	140 (9.76)	1227 (85.5)	207 (14.4)	1212 (84.5)	222 (15.4)	1121 (78.2)
2018 Boonlak^30^	350	Yes	132 (37.7)	59 (36–80)	25.4 ± 4.8	—	99 (75.0)	—	—	84 (63.7)	14 (10.6)	29 (21.9)	5 (3.8)	108 (82.1)
		No	218 (62.2)	58 (31–84)	25.2 ± 4.2	—	162 (74.3)	—	—	127 (58.3)	20 (9.2)	52 (23.8)	19 (8.7)	162 (74.4)
Total	2505	Yes	553	—	—	81 (54)	296 (45.9)	369 (19.3)	52 (21.2)	425 (21.5)	43 (14.2)	204 (13.4)	30 (10)	488 (24.3)
		No	1952	—	—	930 (64.8)	349 (54.1)	1541 (80.7)	193 (78.7)	1547 (78.4)	259 (85.8)	1312 (86.5)	268 (90)	1517 (75.5)

All included studies were judged at “low risk” of bias in the aim, patient selection, data collection, and study endpoint domains. In the endpoint assessment domain, all included studies were judged at “unclear risk” of bias because multivariate analysis for OS was not performed. In the lost to follow‐up domain, all included studies were judged at “unclear risk” of bias because it was not possible to assess if patients lost to follow‐up were less than 5% of the total study population (Figure S2a).

All included studies were suitable for OS analysis, while one study was excluded from DFS analysis because it did not consider this outcome.^29^

**FIGURE 1:**
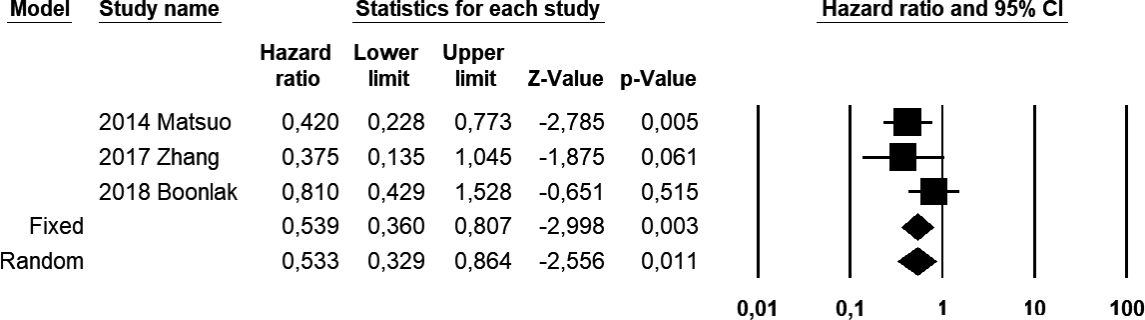
Forest plot of hazard ratios for overall survival of endometrial carcinoma patients with coexistent adenomyosis at univariate analysis; endometrial carcinoma patients without adenomyosis were used as a control group

**FIGURE 2:**
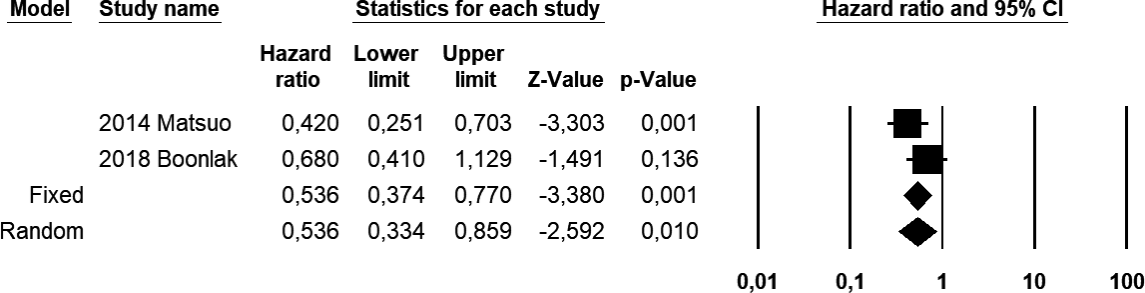
Forest plot of hazard ratios for disease‐free survival of endometrial carcinoma patients with coexistent adenomyosis at univariate analysis; endometrial carcinoma patients without adenomyosis were used as a control group

**FIGURE 3:**
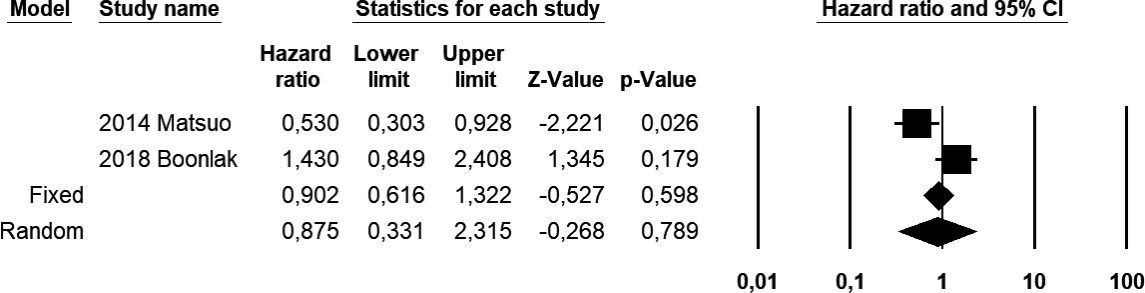
Forest plot of hazard ratios for disease‐free survival of endometrial carcinoma patients with coexistent adenomyosis at multivariate analysis; endometrial carcinoma patients without adenomyosis were used as a control group

Compared with EC patients without adenomyosis, EC patients with coexistent adenomyosis showed a pooled HR of 0.533 (CI 95%, 0.329–0.864; *I* ^2^ = 26.1) for OS at univariate analysis (Figure 1); 0.536 (CI 95%, 0.334–0.859; *I* ^2^ = 41.5) for DFS at univariate analysis (Figure 2); and 0.875 (CI 95%, 0.331–2.315; *I* ^2^ = 84.5) for DFS at multivariate analysis (Figure 3).

## 4 DISCUSSION

The present study shows that EC patients with coexistent adenomyosis have half the risk of death and recurrence compared with EC women without adenomyosis. However, the protective effect of adenomyosis on EC recurrence was not found to be independent from other prognostic factors at multivariate analysis.

Adenomyosis is one of the most frequent findings in hysterectomy specimens from EC patients (up to 70% of cases).^19^, ^20^, ^28^, ^31^ Due to mixed results, the effect of adenomyosis on the prognosis of EC has remained unclear despite its high prevalence. On one hand, coexistent adenomyosis appears to be a risk factor for myometrial invasion, aggressive tumor behavior, and poor prognosis; however, on the other, it appears to be related to good EC prognosis.

Several hypotheses have been proposed to explain the poor prognosis in EC patients with adenomyosis. The large contact area between ectopic endometrium of adenomyosis and the muscular layer might increase the incidence and depth of myometrial infiltration of EC.^22^, ^32^, ^33^ Furthermore, EC might follow the spread mechanism of ectopic endometrium through lymph and veins, increasing lymphovascular space invasion.^22^, ^34^ In addition, the poor prognosis of EC patients with adenomyosis has been associated with the malignant transformation of adenomyosis.^35^, ^36^ Finally, as adenomyosis reduces the contrast between the muscular layers involved in EC and adenomyosis, magnetic resonance imaging shows a decreased diagnostic accuracy in assessing depth of invasion, with potential understaging of patients.^22^, ^37^

In contrast, several other mechanisms might support good prognosis in patients with coexistent adenomyosis. Adenomyosis is characterized by a specific profile of cytokines with increased levels of antitumoral cytokines (such as interferon‐γ, tumor necrosis factor‐α, and interleukin 10) and decreased levels of oncogenic cytokines and growth factors (such as interleukin 1‐beta, interleukin 8, epidermal growth factor, and transforming growth factor). Such a profile of cytokines would lead to alterations in the local microenvironment, limiting tumor progression and invasiveness.^22^, ^34^ In addition, it has also been speculated that the thickened endometrial stroma of the adenomyotic uterus, caused by repeated inflammations with a subsequent healing process, might contribute to a mechanical block of EC invasion in the myometrium.^28^ Finally, the frequent symptoms associated with adenomyosis, such as dysmenorrhea and abnormal uterine bleeding, may lead to an early EC diagnosis and then better cancer prognosis.

Our findings seem to support a positive impact of adenomyosis on the prognosis of women with EC, with the risk of death from any cause halved when compared with EC women without adenomyosis. However, the impact of adenomyosis on OS was only evaluable thorough univariate analysis. In fact, the included studies did not report multivariate analysis for OS, as shown in the risk of bias within studies assessment. Thus, the independent value of coexistent adenomyosis on the risk of death was not evaluable. Further studies with multivariate assessment of the impact of adenomyosis on OS in EC patients are necessary.

In our study, the coexistence of adenomyosis appeared to halve the risk of EC recurrence at univariate analysis; however, this finding was not significant at multivariate analysis. This suggests that this association is not independent. In fact, the association at univariate analysis may depend on other prognostic factors that might be associated with the coexistence of adenomyosis. In this regard, it would be interesting to assess the prevalence of classic prognostic histological factors (i.e. histotype, FIGO grade, deep myometrial infiltration, and lymphovascular space invasion), clinical characteristics impacting prognosis (age, body mass index), and TCGA molecular groups in EC patients with and without adenomyosis in future studies. However, further studies are also required to assess this association as only two included studies allowed data extraction for univariate and multivariate analyses regarding adenomyosis impact on DFS in EC patients.

To the best of our knowledge, this study may be the first systematic review and meta‐analysis to assess the prognostic value of coexistent adenomyosis in patients with EC, without the confounding effect of patient selection based on histological prognostic factors. Moreover, our results are supported by the overall quality of the included studies, as described in the risk of bias assessment. As for limitations, our study appears to be affected by the low number of included studies, in particular regarding DFS analyses. In addition, we were unable to perform OS multivariate analysis since the included studies did not report these. Therefore, we were unable to assess the independent value of adenomyosis on OS.

In conclusion, the risk of death is halved in EC patients with coexistent adenomyosis compared with EC patients without adenomyosis. However, this association needs to be assessed at multivariate analysis in future studies to assess the independence of the prognostic value. Furthermore, adenomyosis does not appear to affect the risk of EC recurrence. Additional data are required to confirm these findings.

## CONFLICTS OF INTEREST

The authors have no conflicts of interest.

## AUTHOR CONTRIBUTIONS

AR, AT: study conception, study design, study methods, data extraction, data analysis, manuscript preparation. DR: study conception, study design, study methods, data analysis, manuscript preparation, methods supervision. MM: study design, study methods, data analysis, manuscript preparation. PC: study conception, study design, study methods, data analysis, manuscript preparation. MA: study conception, data extraction, data analysis, manuscript preparation. ACR: data extraction, data analysis, manuscript preparation. AS: study design, data analysis, manuscript preparation, methods supervision. GZ: study design, methods supervision, whole study supervision. LI, AM, FZ, RS: study conception, study design, methods supervision, whole study supervision.

## Supporting information

Fig S1

Fig S2a

Fig S2b
