# Supplementary figures and images for: Impact of adenomyosis on the prognosis of patients with endometrial cancer
Source: Int J Gynaecol Obstet. 2021 Jul 18;157(2):265–70. doi: 10.1002/ijgo.13818 (PMC9292168; doi:10.1002/ijgo.13818)

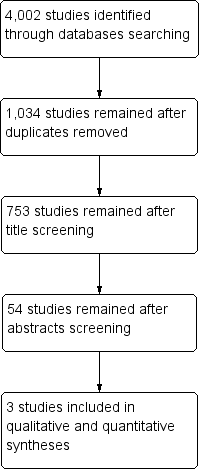

Supplement: Supplementary file 1 — Fig S1 [file IJGO-157-265-s002.png]

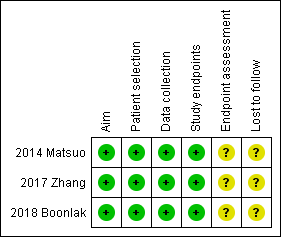

Supplement: Supplementary file 2 — Fig S2a [file IJGO-157-265-s001.png]

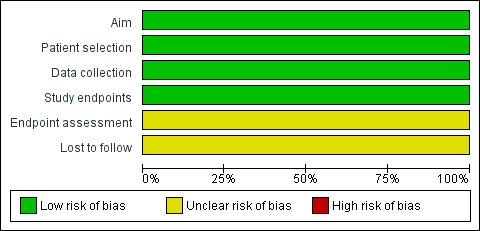

Supplement: Supplementary file 3 — Fig S2b [file IJGO-157-265-s003.png]
